# VII Scandinavian Copd Research Symposium, Holmenkollen, Oslo 18^th^–19^th^ November 2016

**DOI:** 10.1080/20018525.2017.1401867

**Published:** 2017-12-08

**Authors:** A. Lindberg, A. M. Holm, O. Hilberg, T. Harju

**Affiliations:** a Department of Public Health and Clinical Medicine, Unit of Medicine, Umeå University, Umeå, Sweden; b Department of Respiratory Medicine, Oslo University Hospital/University of Oslo, Oslo, Norway; c Department of Medicine, Vejle Hospital, Sygehus Lillebælt, Denmark; d Respiratory Research, Research Unit of Internal Medicine, Medical Research Center Oulu, Oulu University Hospital and University of Oulu, Oulu, Finland

The seventh Scandinavian COPD Research Symposium was held on 18–19 November 2016 at Scandic Holmenkollen Park Hotel, Oslo, Norway. Since the first meeting in 2004, the purpose of these Symposia have been to allow young researchers from Denmark, Finland, Norway and Sweden to present their research, thereby also facilitating networking, collaboration and stimulating future research in the field of COPD. Ten young scientists from our countries presented their research, and three state-of the art lectures covered the areas Biomarkers and Inflammation in COPD, E-cigarettes and COPD, and Imaging in COPD. Within the topics of each state-of-the-art the lecturers held in-depth discussions with the participants on the second day of the meeting. The meeting was generously supported by grants from Boehringer Ingelheim, which also made publication of this supplement to *European Clinical Respiratory Journal* possible.

## State-of-the art abstracts

### Biomarkers and inflammation in COPD

Jörgen Vestbo, Copenhagen, Denmark

Professor Jörgen Vestbo

Division of Infection,

Immunity and Respiratory Medicine,

University of Manchester, UK

jorgen.vestbo@manchester.ac.uk

There is a need for biomarkers in COPD – in order to evaluate disease activity, to characterise disease phenotypes and endotypes and to evaluate risk of exacerbations and complications such as comorbidities. Not surprisingly, markers of systemic inflammation have been the target of research as COPD is seen as a disease where inflammation has been part of the definition until now and where easily accessible biomarkers are available.

The main biomarkers studied have been fibrinogen hs-CRP, white blood cell count and differential count (eosinophils), as well as by-products resulting from inflammation, not least markers of matrix remodelling and breakdown. Most studies have shown that markers of systemic inflammation have independent prognostic value regarding exacerbations, hospitalisation and mortality, although studies with fairly dubious associations can be found. As a result of studies on fibrinogen, the US Food and Drug Administration (FDA) has decided to approve fibrinogen as the first biomarker in COPD for enriching controlled trials using exacerbations as primary outcome. None of these markers of systemic inflammation seem to reflect ongoing disease activity (when measured as FEV1 decline), but design of studies of FEV1 decline have inherent biases as a result of the variety of trajectories that can lead to clinical COPD. Many of the associations found may also reflect the well-known relationship between smoking and systemic inflammation. Lately, eosinophils have been seen as ‘the new black’ in COPD research. Fairly limited prospective data exist to evaluate the value of eosinophils in predicting response to inhaled (and systemic) corticosteroids in COPD. The issue of determining relevant cut-offs is also unresolved.

Systemic inflammation is now known to be present in a subset of COPD patients, to be associated with poor prognosis, but is not associated with subsequent FEV1 decline. Other biomarkers may have larger potential in this respect. Overall, this research field is still fairly open.

### E-cigarettes and COPD

Robert Foronjy, New York, USA

Robert Foronjy, M.D.

Visiting Associate Professor of Medicine

Chief, Pulmonary and Critical Care Medicine

SUNY Downstate Medical Center, MSC 19

Brooklyn, New York 11203

robert.foronjy@downstate.edu

**Background**: Public health officials have supported the use of electronic (e)-cigarettes to reduce harm amongst nicotine-addicted cigarette users. However, the lung health effects of e-cigarettes remain unknown and long-term clinical studies in humans will take decades to complete. To assess their impact in a more timely manner, this study exposed mouse lung and normal human airway epithelial cells to aerosolised nicotine-free and nicotine-containing e-cigarette fluid.

**Methods**: Mice were exposed to aerosolised phosphate-buffered saline, nicotine-free or nicotine-containing e-cigarette solution, 1 h daily for 4 months. Normal human bronchial epithelial (NHBE) cells cultured at an air–liquid interface were exposed to e-cigarette vapours or nicotine solutions using a Vitrocell smoke exposure robot.

**Results**: Chronic exposure to nicotine-containing e-cigarette aerosol increased airway hyper-reactivity, distal airspace enlargement, mucin production, cytokine and protease expression. Inhalation of nicotine-free e-cigarette aerosol had no effect on these lung parameters. NHBE cells exposed to nicotine-containing e-cigarette vapour showed impaired ciliary beat frequency, airway surface liquid volume, cystic fibrosis transmembrane regulator and ATP-stimulated K+ ion conductance and decreased expression of FOXJ1 and KCNMA1. Exposure of NHBE cells to nicotine for 5 days increased interleukin (IL)-6 and IL-8 secretion.

**Conclusions**: Chronic exposure to nicotine-containing e-cigarette fluids induces COPD associated parameters including cytokine expression, airway hyper-reactivity and lung tissue destruction. These responses were nicotine-dependent both in the mouse lung and in human airway cells, indicating that inhaled nicotine contributes to airway and lung disease in addition to its addictive properties. Thus, this study underscores the potential dangers of chronic nicotine inhalation during e-cigarette use.

### Imaging in COPD

Terttu Harju, Oulu, Finland

Professor Terttu Harju

Respiratory Research, Research Unit of Internal Medicine,

Medical Research Center Oulu,

Oulu University Hospital and University of Oulu,

Oulu, Finland

terttu.harju@oulu.fi

Chronic obstructive pulmonary disease (COPD) is a heterogenous disease with different clinical phenotypes due partly to differences in morphologic abnormalities. CT is not routinely recommended. However, emphysema, airway disease, air trapping and pulmonary vascular abnormalities have all been associated with a number of important outcomes, including respiratory symptoms, COPD exacerbations and mortality. A more precise definition of COPD phenotype may be helpful in personalised treatment [1]. Small airway disease is an important major component of both emphysema-predominant disease and airway-predominant disease involving larger airways (bronchi). Isolated small airway disease can also occur as a primary expression of COPD and CT can be helpful in identifying signs of inflammatory small airway disease and small airway obstruction. CT is also needed to identify interstitial abnormalities likely corresponding to variable combinations of respiratory bronchiolitis, airspace enlargement with fibrosis, and smoking-related interstitial fibrosis. Combined pulmonary fibrosis and emphysema has worse prognosis and more pulmonary hypertension than phenotypes with no fibrosis. Pulmonary hypertension can be identified by CT enlargement of the pulmonary artery as determined by a pulmonary artery–aorta ratio of more than 1, and was recently shown to be an independent risk factor for COPD exacerbations. CT measurements of emphysema are considered key surrogate endpoints in a1-antitrypsin deficiency clinical trials. CT has become a critical measurement tool for morphologic phenotyping in large-scale cohort studies. Pulmonary imaging techniques such as functional CT and MRI are used in research, to visualise and quantify regional improvements in ventilation over time in response to COPD therapy, even in the absence of clinically relevant changes in FEV1, and to study regional gas trapping and airway morphology as well as in new interventions for improving regional airflow.

### 

Reference1.
LynchDA, AustinJHM, HoggJC, et al
CT-definable subtypes of chronic obstructive pulmonary disease: a statement of the Fleischner Society. Radiology. 2015;277:1–8.10.1148/radiol.2015141579PMC461387825961632

## Abstracts from other lectures

### Ultrastructural and contraction properties of myofibroblasts in nonsmokers, smokers and COPD

Ninni-Ingrid Nurmos, University of Oulu, Finland

Ninni-Ingrid Nurmos^1^, Henna Karvonen^1,2^, Elisa Lappi-Blanco^1,3,4^, Terttu Harju^1^, Magnus Sköld^5^, Siri Lehtonen^1^, and Riitta Kaarteenaho^1,6^

^1^Respiratory Research, Research Unit of Internal Medicine, Medical Research Center Oulu, Oulu University Hospital and University of Oulu, Oulu, Finland, ^2^Laboratory of Tissue Repair and Regeneration, Matrix Dynamics Group, Faculty of Dentistry, University of Toronto, Canada, ^3^Department of Pathology, Oulu University Hospital, Oulu, Finland, ^4^Department of Pathology, Cancer and Translational Medicine Research Unit, University of Oulu, Finland, ^5^Respiratory Medicine Unit, Center for Molecular Medicine, Department of Medicine Solna, Karolinska Institutet; Lung Allergy Clinic, Karolinska University Hospital Solna, Stockholm, Sweden, ^6^Unit of Medicine and Clinical Research, Pulmonary Division, School of Medicine, Faculty of Health Sciences, University of Eastern Finland; Center of Medicine and Clinical Research, Division of Respiratory Medicine, Kuopio University Hospital, Kuopio, Finland.

ninni-ingrid.nurmos@student.oulu.fi

Fibroblasts and myofibroblasts are key stromal cells in fibrotic processes in chronic obstructive pulmonary disease (COPD).

Our aim was to compare ultrastructural and functional properties of stromal cells isolated from central and peripheral lung of nonsmokers, smokers and individuals with COPD. Stromal cells were collected and cultured from patients undergoing lung cancer surgery. According to the clinical and lung function data, the patients were classified as nonsmokers, smokers with normal lung function and COPD. Myofibroblasts were investigated by transmission electron microscopy (TEM) and immunoelectron microscopy (IEM). Functional properties of cells were analysed by three-dimensional collagen gel contraction assay. Expression of alpha smooth muscle actin (α-SMA), a marker for myofibroblasts, was measured by Western blotting. Central myofibroblasts from patients with COPD showed more frequently tandem-like fibronexi, specific ultrastructures of myofibroblasts, compared to others. More labels for α-SMA were observed in central than in peripheral myofibroblasts in individuals without COPD. Stromal cells from peripheral lung contracted more than those from central lung, and cells of smokers contracted more than those of nonsmokers. Peripheral cells of smokers with or without COPD expressed lower levels of α-SMA compared to non-smokers. Central fibroblasts of smokers without COPD expressed less α-SMA than fibroblasts of nonsmokers.

We concluded that properties of lung fibroblasts and myofibroblasts vary depending on the anatomical origin as well as on smoking and COPD; these phenomena need to be taken into account when designing research protocols and drug testing for COPD.

### Spirometric criteria and reference values – consequences on COPD prevalence

Helena Backman, Umeå University, Sweden

Department of Public Health and Clinical Medicine, Occupational and Environmental Medicine, Umeå University, Umeå, Sweden

helena.backman@norrbotten.se

Lung function is an extremely important measure in the evaluation of COPD, and defining normality is important, both for diagnosis and classification of severity. The definition of COPD has changed over time, and the definition of a decreased FEV_1_/(F)VC ratio is still under debate.[1–3] Two commonly used definitions today are the GOLD [4] and ERS/ATS [1] definitions of a post-bronchodilator FEV_1_/FVC < 0.7 and FEV1/FVC < LLN (lower limit of normal), respectively, where the LLN is set by the choice of reference values for spirometry. Simplicity is the main argument for the fixed ratio criterion advocated by GOLD,[4,5] while the ERS/ATS points out under-diagnosis among younger subjects and over-diagnosis among elderly associated with the fixed ratio criterion.[1,3] The GOLD criterion has been most frequently used, especially in the clinics, while the LLN criterion is gaining ground in epidemiological studies on COPD. Large population-based studies including spirometry are the preferred methods to estimate the prevalence of COPD in a population. Usually, the prevalence is based on spirometry results, without taking symptoms and exposures into account, and the identified subjects are classified by disease severity by the level of their FEV_1_ expressed as % of the corresponding reference value. The prevalence of COPD is markedly affected by the choice between slow or forced vital capacity, pre- or post-bronchodilator spirometry, fixed ratio or LLN as cut-off, and the choice of reference values.

#### 

References1.
PellegrinoR, ViegiG, BrusascoV, et al
Interpretative strategies for lung function tests. Eur Respir J. 2005
11;26(5):948–968.1626405810.1183/09031936.05.000352052.
CelliBR, Halbert RJ. Point: should we abandon FEV(1)/FVC <0.70 to detect airway obstruction? No. Chest. 2010
11;138(5):1037–1040.10.1378/chest.10-2049210513933.
EnrightP, Brusasco V. Counterpoint: should we abandon FEV(1)/FVC < 0.70 to detect airway obstruction? Yes. Chest. 2010
11;138(5):1040–1042; discussion
1042–1044.10.1378/chest.10-2052210513944.
The global initiative for chronic obstructive lung disease (GOLD)
2015
[cited 2015 Nov 27]
Available from: www.goldcopd.org
5.
VestboJ, HurdSS, AgustíAG, et al
Global strategy for the diagnosis, management, and prevention of chronic obstructive pulmonary disease: GOLD executive summary. Am J Respir Crit Care Med. 2013
2
15;187(4):347–365.2287827810.1164/rccm.201204-0596PP

### Symptom experiences in patients with chronic obstructive pulmonary disease

Vivi Lycke Christensen, University of Oslo, Norway

V. L. Christensen^1^, T. Rustøen^2^, B. A. Cooper^3^, C. Miaskowski^4^, A. H. Henriksen^5^, S. B. Bentsen^6^, and A. M. Holm^7^

^1^Division of Emergencies and Critical Care, Department of Research and Development, Oslo University Hospital, Ullevål; Institute of Clinical Medicine, Faculty of Medicine, University of Oslo; Lovisenberg Diaconal University College, ^2^Institute of Clinical Medicine, Faculty of Medicine, University of Oslo; Department of Nursing Science, Institute of Health and Society, University of Oslo, Oslo, Norway, ^3^Department of Community Health Systems, ^4^Department of Physiological Nursing, University of California, San Francisco, CA, USA, ^5^Department of Circulation and Medical Imaging, St Olav’s University Hospital, Trondheim, ^6^Department of Health Studies, University of Stavanger, Stavanger, ^7^Institute of Clinical Medicine, Faculty of Medicine, University of Oslo; Department of Respiratory Medicine, Oslo University Hospital, Rikshospitalet, Oslo, Norway

v.l.christensen@medisin.uio.no

Patients with chronic obstructive pulmonary disease (COPD) experience multiple co-occurring symptoms. Previous research has mostly focused on these patients’ symptom experiences in the advanced stages of disease.

The aims of this study were to evaluate for differences in symptom occurrence rates among patients (*n* = 267) with moderate, severe, and very severe COPD, to identify subgroups of patients with distinct symptom experiences, and to determine if these subgroups differed on patient characteristics and disease-specific quality of life (QOL).

The Memorial Symptom Assessment Scale (MSAS) was used to evaluate the patients’ symptom experiences based on the occurrence rates of self-reported symptoms. Binary logistic regression with stage of disease as an ordinal predictor variable was used to evaluate for differences in symptom occurrence rates. Latent class analysis (LCA) was used to identify subgroups of patients with distinct symptom experiences.

Regardless of severity of disease, patients reported an average of 12 co-occurring symptoms. Three subgroups of patients were identified and named ‘high’, ‘intermediate’, and ‘low’. Patients in the ‘high’ group had the highest occurrence rates for psychological symptoms. These patients were younger, more likely to be women, had significantly more acute exacerbations in the past year, and reported significantly worse disease-specific QOL scores.

Findings from this study suggest that patients with COPD experience a high burden of multiple symptoms regardless of severity of disease. Patients with a higher symptom burden may have therapeutic needs that may not be identified using current classifications that are based only on respiratory function.

### Physical activity and physical capacity in subjects with chronic obstructive pulmonary disease

Mikael Andersson, Uppsala University, Sweden

Mikael Andersson^1,2^

^1^Department of Neuroscience, Physiotherapy, Uppsala University, Uppsala, Sweden, ^2^Department of Medical Science, Respiratory Medicine and Allergology, Uppsala University, Uppsala, Sweden

mikael.s.andersson@neuro.uu.se

Reduced physical activity levels are common in subjects with COPD and are associated with increased morbidity and mortality.[1] Pulmonary rehabilitation should therefore be offered to subjects with a low physical activity.

As part of my doctoral thesis we have investigated factors associated with low physical activity levels both in a selective sample of patients (*n* = 73) as well as in a sample of subjects with (*n* = 403) and without COPD (*n* = 659) from the population-based OLIN COPD study.[2]

In the selective sample,[3] using an objective method for assessing physical activity, 92% were classified as physically inactive or sedentary. About 45% of the variance in physical activity levels were explained by lung function (22%), walking speed (10%), muscle strength (7%), and fat-free mass (3%). Age, gender and systemic inflammation did not contribute to the statistical model.

In the population-based sample,[4] using questionnaires to assess physical activity levels, the prevalence of low physical activity was higher among subjects with COPD than without (22.6% vs. 14.6%, respectively). In subjects with COPD, the factors most strongly associated with low physical activity were older age, OR 1.52 (95% CI 1.12–2.06), a history of heart disease, OR 2.11 (1.10–4.08), and clinically significant fatigue, OR 2.33 (1.31–4.13).

Among non-COPD subjects, obesity was the only significant factor, OR 2.26 (1.17–4.35).

Our results indicate that attentiveness to patients’ physical function as well as to symptoms of fatigue and concomitant heart disease may be useful in identifying subjects in need of pulmonary rehabilitation.

### 

References1.
WatzH, PittaF, RochesterCL, et al
An official European Respiratory Society statement on physical activity in COPD. Eur Respir J. 2014;44(6):1521–1537.2535935810.1183/09031936.000468142.
LindbergA, LundbäckB.
The obstructive lung disease in northern Sweden chronic obstructive pulmonary disease study: design, the first year participation and mortality. Clin Respir J. 2008
10;2(Suppl 1):64–71.2029835210.1111/j.1752-699X.2008.00086.x3.
AnderssonM, SlindeF, GrönbergAM, et al
Physical activity level and its clinical correlates in chronic obstructive pulmonary disease: a cross-sectional study. Respir Res. 2013
11
15;14(1):128.2423787610.1186/1465-9921-14-128PMC38326834.
AnderssonM, StridsmanC, RönmarkE, et al
Physical activity and fatigue in chronic obstructive pulmonary disease – a population based study. Respir Med. 2015
8;109(8):1048–1057.2607027210.1016/j.rmed.2015.05.007

### Physical activity and prognosis in COPD patients

Marie J. Waatevik, Haukeland University Hospital, Norway

Marie Waatevik^1^, Ane Johannessen^2^, Francisco Gomez Real^3,4^, Marianne Aanerud^5^, Jon Andrew Hardie^3^, Per Sigvald Bakke^3^, and Tomas Mikal Lind Eagan^3,5^

^1^Centre for Clinical research, Haukeland University Hospital, Bergen, Norway, ^2^Centre for International Health, Department of Global Public Health and Primary Care, University of Bergen, Bergen, Norway, ^3^Department of Clinical Science, University of Bergen, Bergen, Norway, ^4^Department of Gynecology and Obstetrics, Haukeland University Hospital, Bergen, Norway, ^5^Department of Thoracic Medicine, Haukeland University Hospital, Bergen, Norway

marie.waatevik@helse-bergen.no

The use of six-minute walk test (6MWT) to measure exercise capacity is a valuable method in both treatment and examination of COPD patients. The 6MWT provides information on walking distance, oxygen desaturation and dyspnoea.

The aim of this project is to examine how physical activity and exercise capacity (measured through the 6MWT), and the functional responses to those, impact COPD prognosis. We focus on oxygen desaturation during 6MWT as a predictor for important COPD outcomes: mortality, frequency of exacerbations, decline in lung function, and decline in lean body mass.

A total of 433 COPD patients were included in the Bergen COPD Cohort Study 2006–2009, and followed up for 3 years. Patients were characterised with spirometry, bioelectrical impedance measurements, Charlson co-morbidity score, exacerbation history, smoking, and arterial blood gases. 370 patients completed the 6MWT at baseline of the study. Information on all-cause mortality was collected in 2011.

Patients who experienced oxygen desaturation during the 6MWT had more than twofold increased risk of death; hazard ratio (95% CI) 2.4 (1.2, 5.1), a 50% increased risk for COPD exacerbations; incidence rate ratio (95% CI) 1.6 (1.1, 2.2), double the rate of decline in both FVC and FEV1 (3.3% and 1.7% versus 1.7% and 0.9% respectively), and manifold increased rate of loss of lean body mass (0.18 kg m^–^^2^ versus 0.03 kg m^–^^2^ among those who did not desaturate).[1]

COPD patients that desaturated had significantly worse prognosis than non-desaturating COPD patients, for multiple important disease outcomes.[1]

Reference1.
WaatevikM, JohannessenA, RealF, et al
Oxygen desaturation in 6-min walk test is a risk factor for adverse outcomes in COPD. Eur Respir J. 2016;48:82–91.2707658610.1183/13993003.00975-2015

### Mindfulness-based cognitive therapy (MBCT) in COPD: preliminary results of a randomised controlled trial

Ingeborg Farver-Vestergaard, Aarhus University Hospital, Denmark

Ingeborg Farver-Vestergaard^1^, Anders Løkke Ottesen^2^, Elisabeth Bendstrup^2^, Maja O’Connor^1^, Robert Zachariae^1^

^1^Unit for Psychooncology and Health Psychology, Aarhus University and Aarhus University Hospital, Aarhus, Denmark, ^2^Department of Respiratory Diseases and Allergy, Aarhus University Hospital, Aarhus, Denmark

ifarver@psy.au.dk

**Background**: Symptoms of anxiety and depression are prevalent in COPD and are associated with a higher symptom burden, increased health care utilisation and lower quality of life.[1] Psychosocial intervention may be an effective supplement to already established treatment regimens in COPD.[2]

**Aim**: We conducted a randomised controlled trial testing the effect of a standardised 8-week mindfulness-based cognitive therapy (MBCT) programme [3] on anxiety, depression and disease-specific quality-of-life in COPD.

**Methods**: A total of 84 patients with severe to very severe COPD (age = 67.18 (7.79); women = 57.1%) were randomised to an intervention group (*n* = 39), receiving MBCT as an add-on to a standardised 8-week pulmonary rehabilitation (PR) programme, or a control group (*n* = 45), receiving PR only. Participants completed questionnaires (the Hospital Anxiety and Depression Scale (HADS); the COPD Assessment Test (CAT)) before and after the intervention.

**Results**: On average, patients in the intervention group attended 4.08 (SD = 2.74) out of eight MBCT sessions. The results of a time × group mixed between-within subjects factorial ANOVA revealed a significant main interaction effect (F(1,64) = 5.714; *p* = 0.020) on HADS scores, corresponding to a medium effect size (ηp^2^ = 0.082) and a clinically important mean difference in the intervention group (>1.5). The main interaction effect on CAT scores did not reach statistical significance (F(1,30) = 1.095, *p* = 0.304, ηp^2^ = 0.035).

**Conclusion**: MBCT may be an effective intervention for reducing anxiety and depression in COPD. Tele-based formats of delivery could be considered in the future with the purpose of minimising missing data and patient non-attendance.

### 

References1.
MaurerJ, RebbapragadaV, BorsonS, et al
Anxiety and depression in COPD: current understanding, unanswered questions, and research needs. Chest. 2008;134(4):43–56.10.1378/chest.08-0342PMC2849676188429322.
Farver-VestergaardI, JacobsenD, ZachariaeR.
Efficacy of psychosocial interventions on psychological and physical health outcomes in chronic obstructive pulmonary disease: a systematic review and meta-analysis. Psychother Psychosom. 2015;84(1):37–50.2554764110.1159/0003676353.
SegalZV, WilliamsJMG, TeasdaleJ.
Mindfulness-based cognitive therapy for depression. New York (NY): Guildford Press; 2013.

### Elevated sputum vitamin D binding protein levels in heavy smokers with chronic obstructive pulmonary disease

Tanja Törölä, University of Helsinki, Finland

T. Törölä^1^, P. Nieminen^2^, S. Ohlmeier^3^, J. Gao^1^, T. Toljamo^4^, W. Mazur^1^, and V. Pulkkinen^1^

^1^University of Helsinki, Helsinki University Hospital, Heart and Lung Center, Department of Pulmonary Medicine, Helsinki, Finland, ^2^University of Oulu, Medical Informatics and Statistics Group, Oulu, Finland, ^3^Biocenter Oulu, Proteomics Core Facility, University of Oulu, Faculty of Biochemistry and Molecular Medicine, Oulu, Finland, ^4^Lapland Central Hospital, Department of Pulmonary Medicine, Rovaniemi, Finland.

tanja.torola@hus.fi

In our previous proteomic screening study we showed that sputum vitamin D binding protein (VDBP) was elevated in chronic obstructive pulmonary disease (COPD).[1] Interestingly, VDBP encoding gene (GC) variants have been associated with COPD and vitamin D deficiency.[2] The aim of the current study was to examine the association of sputum VDBP as well as circulating VDBP and vitamin D with changes of lung function over a 4-year follow-up. The biomarker levels and the GC rs4588 and rs7041 genotypes were assessed in non-smokers (*n* = 34), heavy smokers without COPD (*n* = 158) as well as in smokers with COPD GOLD 2007 stage I (*n* = 22) and stage II–III (*n* = 27). Sputum VDBP levels were elevated in COPD patients compared to non-smokers and in COPD stage II–III compared to smokers without COPD. Furthermore, elevated sputum VDBP significantly correlated with symptoms, quality of life, and a faster decline of lung function (FEV1 % predicted and FEV1/FVC %) over 4 years in patients with COPD stage I. In regression analysis COPD and smoking were the strongest predictors of elevated sputum VDBP. Plasma VDBP levels did not differ between the study groups or correlate with lung function, but had a weak positive correlation with vitamin D levels. Plasma VDBP levels were dependent on the GC rs7041 genotype, though it was not a confounding factor for sputum VDBP levels. In conclusion, elevated sputum VDBP may indicate a faster decline of lung function in COPD. GC gene genotypes should be considered when evaluating VDBP as a potential biomarker of COPD.

#### 

References1.
OhlmeierS, MazurW, Linja-AhoA, et al
Sputum proteomics identifies elevated PIGR levels in smokers and mild-to-moderate COPD. J Proteome Res. 2012;11:599–608.2205382010.1021/pr20063952.
JanssensW, BouillonR, ClaesB, et al
Vitamin D deficiency is highly prevalent in COPD and correlates with variants in the vitamin D-binding gene. Thorax. 2010;65(3):215–220.1999634110.1136/thx.2009.120659

### Hypogammaglobulinaemia in COPD patients

Siw Lillevik Andreassen, Oslo University Hospital, Norway

Siw L. Andreassen^1^, Vivi L. Christensen^2^, Tone Rustøen^2^, Olav Klingenberg^2^, Ingebord Aarberge^3^, Christine Miaskowski^4^, and Are M. Holm^5,6^

^1^Internal Medicine, Drammen Sykehus, ^2^Oslo University Hospital, Ullevål, ^3^Folkehelseinstituttet, Oslo, ^4^University of California, San Francisco (UCSF), USA, ^5^Oslo University Hospital, Rikshopsitalet, ^6^University of Oslo (UiO)

siwlille.andeassen@gmail.com

COPD is a complex disease, and there is evidence to define at least three different phenotypes: overlap or mixed COPD-asthma, exacerbator, and emphysema-hyperinflation. In particular, patients with frequent exacerbation have a high mortality and rapid disease progression. There may be several reasons why some COPD patients have frequent exacerbations, both in genetics, airway damage and the immune system. The aim of this study is to determine the prevalence of hypogammaglobulinaemia in a cohort of stable COPD patients and to relate immunoglobulin levels to manifestations of COPD, such as lung function, frequency of exacerbations and self-reported symptoms. Our study project, symptom clusters and immune markers in patients with COPD, included patients with stable COPD, GOLD II–IV, in an outpatient clinic at hospitals in the south-eastern area of Norway. Lung function tests, questionnaires, blood tests and clinical tests were registered, and also at a one-year follow up. In total 267 patients were included, of which 30 patients (11.5%) had IgG < 6.1. These patients were defined as hypogamma-COPD. Subjects with hypogamma-COPD were compared to the other COPD patients. In this group, patients had lower FEV1 and a higher proportion had GOLD stage IV; they also had a higher number of COPD admissions, and treatment cures with antibiotics and prednisolone in the last year. Moreover, patients with hypogamma-COPD did not have a higher occurrence of chronic bronchitis or variable obstruction. There was no significant difference in the frequency of emphysema. Importantly, we found that hypogamma-COPD was related to the occurrence of acute exacerbations in the preceding year, as well as a severe COPD grade.

### New approaches of lung function measurements in COPD

Linnea Jarenbäck, University of Lund, Sweden

Respiratory medicine and Allergology, Lund University

linnea.jarenback@med.lu.se

There are different phenotypes of COPD, e.g. bronchitis, type of emphysema, bronchodilator responders and frequent exacerbators. It is important to characterise and clinically phenotype each individual, to aid the development of more individualised therapies.

At the Lund University Hospital respiratory research unit, we investigated baseline lung physiology parameters and reversibility pattern using advanced lung function measurements (spirometry, body plethysmography, impulse oscillometry, CO-diffusion and multiple breath washout of N2) in smoking controls and COPD patients. Our aim was to gain further insight into the disease development and progression. Furthermore, we aimed to improve respiratory care for COPD patients, by identifying parameters important for disease development, progression and therapy response.

In June 2016, my thesis: ‘COPD- lung physiology and genetic links’ was published. Data presented here comprise the lung physiology section of this thesis. Our main findings included: (1) FEV1%p did not correlate with other lung function parameters within each GOLD stage; (2) several parameters showed segmented relationships over a linear relationship to FEV1%p, and had break points where the disease pattern changed at FEV1%p 60–70; (3) COPD patients showed increased ventilation heterogeneity in the acinar airways which was associated with decreased diffusion capacity, but no response to bronchodilators; and (4) reversibility of other lung function parameters did not always correlate to the reversibility in FEV1%p, thus separating some flow responders from volume or resistance responders.

### Is singing efficient in COPD rehabilitation?

Mette Kaasgaard, Aarhus University Hospital, Denmark

Aarhus University Hospital, Pulmonary Department and Center for Music in the Brain, Clinical Department, Aarhus University, Aarhus, Denmark

mette2706@gmail.com

After diagnosis, COPD patients are offered participation in pulmonary rehabilitation (PR) for the purpose of better managing disease and symptoms and for avoiding future relapses and hospitalisations. However there are a large number of dropouts from the PR programme, and a need for investigation of alternative activities. We suggest group singing as such potential relevant and motivating activity. Internationally singing therapy (ST) for COPD patients has been explored with positive indications, and in Denmark it has become common-sense that singing is healthy to COPD patients. However the field has not yet been investigated in a Danish context.

We aim to examine the effects of 10-week intervention based group singing. The main hypothesis of this study is that singing might lead to a positive and consistent rehabilitation process which regards physiological as well as psychological and psychosocial parameters compared to current PR.

The study is a mixed method study, primarily based on a randomised controlled trial with three groups: singing, singing and physical training, and an active control group receiving physical training. Qualitative studies are also part of the project. We aim to include 240 patients.

Group singing potentially leads to improvements on individual patient level and to higher motivation in relation to the rehabilitation programme. Also ST might lead to less consumption of medicine, a decrease in exacerbations and hospitalisations, and as a result a decrease in national health care expenses. In case of successful findings ST could therefore be considered accepted within the national standard PR programme.

## References

[1] Lynch DA, Austin JHM, Hogg JC, et al CT-definable subtypes of chronic obstructive pulmonary disease: a statement of the Fleischner Society. Radiology. 2015;277:1–8. 10.1148/radiol.2015141579 PMC4613878 25961632

